# Reciprocal mutations of lung-tropic AAV capsids lead to improved transduction properties

**DOI:** 10.3389/fgeed.2023.1271813

**Published:** 2023-11-22

**Authors:** Ashley L. Cooney, Christian M. Brommel, Soumba Traore, Gregory A. Newby, David R. Liu, Paul B. McCray, Patrick L. Sinn

**Affiliations:** ^1^ University of Iowa, Stead Family Department of Pediatrics, Iowa City, IA, United States; ^2^ Pappajohn Biomedical Institute, Iowa City, IA, United States; ^3^ Center for Cystic Fibrosis Gene Therapy, University of Iowa, Iowa City, IA, United States; ^4^ Merkin Institute of Transformative Technologies in Healthcare, Broad Institute of Harvard and MIT, Cambridge, MA, United States; ^5^ Department of Chemistry and Chemical Biology, Harvard University, Cambridge, MA, United States; ^6^ Howard Hughes Medical Institute, Harvard University, Cambridge, MA, United States

**Keywords:** adeno-associated virus capsid, gene therapy, cystic fibrosis, airway epithelia, base editing

## Abstract

Considerable effort has been devoted to developing adeno-associated virus (AAV)-based vectors for gene therapy in cystic fibrosis (CF). As a result of directed evolution and capsid shuffling technology, AAV capsids are available with widespread tropism for airway epithelial cells. For example, AAV2.5T and AAV6.2 are two evolved capsids with improved airway epithelial cell transduction properties over their parental serotypes. However, limited research has been focused on identifying their specific cellular tropism. Restoring cystic fibrosis transmembrane conductance regulator (*CFTR*) expression in surface columnar epithelial cells is necessary for the correction of the CF airway phenotype. Basal cells are a progenitor population of the conducting airways responsible for replenishing surface epithelial cells (including secretory cells and ionocytes), making correction of this cell population vital for a long-lived gene therapy strategy. In this study, we investigate the tropism of AAV capsids for three cell types in primary cultures of well-differentiated human airway epithelial (HAE) cells and primary human airway basal cells. We observed that AAV2.5T transduced surface epithelial cells better than AAV6.2, while AAV6.2 transduced airway basal cells better than AAV2.5T. We also investigated a recently developed capsid, AAV6.2FF, which has two surface tyrosines converted to phenylalanines. Next, we incorporated reciprocal mutations to create AAV capsids with further improved surface and basal cell transduction characteristics. Lastly, we successfully employed a split-intein approach using AAV to deliver an adenine base editor (ABE) to repair the *CFTR* ^R553X^ mutation. Our results suggest that rational incorporation of AAV capsid mutations improves AAV transduction of the airway surface and progenitor cells and may ultimately lead to improved pulmonary function in people with CF.

## 1 Introduction

Adeno-associated virus (AAV)-based vectors have been evaluated in five Phase I and II clinical trials for cystic fibrosis. In the early to mid-2000s, aerosolized AAV2 was tested in over 100 trial participants and determined to be safe, but no significant improvements in lung function were reported (Moss et al., 2004; Moss et al., 2007). The vectors in these trials used the AAV2 capsid and relied on the weak ITR promoter to drive expression of the ∼4.5 kb cystic fibrosis transmembrane conductance regulator (*CFTR*) cDNA. The suboptimal AAV2 serotype and the absence of a strong exogenous promoter likely resulted in the lack of clinical improvements. With the goal of developing a gene therapy reagent for cystic fibrosis (CF), many strategies to improve AAV vector transduction and gene expression in airway epithelial cells have been advanced, such as improved capsid design (Excoffon et al., 2009; Limberis et al., 2009), a short but strong promoter (Yan et al., 2015), shortened *CFTR* cDNA (Ostedgaard et al., 2005), and incorporating proteasome modulators to increase AAV genome trafficking to the nucleus (Zhang et al., 2004). Each of these strategies has improved AAV transduction and transgene expression in airway cells. Indeed, such advances in AAV vector design led to the demonstrated complementation of CFTR anion channel function in a CF pig model (Steines et al., 2016; Cooney et al., 2019). Importantly, new AAV capsid designs are currently undergoing clinical trials at 4D Molecular Therapeutics (https://apps.cff.org/Trials/Pipeline/details/10197/4D-710), and an additional AAV clinical trial by Spirovant (https://apps.cff.org/Trials/Pipeline/details/10160/Spirovant-Sciences) is anticipated in the near term.

To improve the AAV tropism of airway epithelial cells, multiple groups have described novel capsids. For example, AAV6.2 (Limberis et al., 2009) was generated by incorporating an F129L point mutation in the AAV6 capsid (Halbert et al., 2001). Likewise, AAV2.5T resulted from directed evolution in airway cells and is comprised of portions of the AAV2 (Rabinowitz et al., 2002) and AAV5 serotypes (Chiorini et al., 1999) with a point mutation at position A581T (Excoffon et al., 2009; Dickey et al., 2011). These modifications reportedly enhanced entry by 100-fold over their parental serotypes (Excoffon et al., 2009; Dickey et al., 2011). The goal of this study was to modify reciprocal residues from AAV6.2 and AAV2.5T to further improve the transduction of surface airway epithelial cells.

After cell entry, surface tyrosine residues of the AAV capsid can be ubiquitinated, causing the vector to be shuttled to the proteasome for degradation (Zhong et al., 2008a). We and others observed that proteasome inhibitors increase transgene expression from AAV vectors (Ding et al., 2003; Mitchell and Samulski, 2013). In the absence of proteasome inhibitors, there is reduced transgene expression, possibly due to ubiquitin/proteasome interactions that may prevent AAV trafficking to the nucleus (Zhang et al., 2004). The introduction of tyrosine (Y) to phenylalanine (F) mutations in the AAV6.2 capsid at positions Y445 and Y731 (termed AAV6.2FF (van Lieshout et al., 2018)) increased transgene expression, possibly by preventing phosphorylation of tyrosine residues (Kang et al., 2020). Reducing or eliminating the need for proteasome inhibitors would be advantageous for clinical applications. In this study, we investigate whether mutating analogous surface Y residues of AAV2.5T to F similarly increases airway epithelial cell transduction. We show that the “FF” mutants still require proteasome modulation but showed increased transgene expression relative to AAV2.5T at all doxorubicin (Dox) concentrations tested.

AAVs are used to deliver gene editing tools such as CRISPR-based adenine base editors (ABEs). A Cas9 nickase is fused to an evolved adenosine deaminase to convert adenine nucleotides to guanine nucleotides through an inosine intermediate (Levy et al., 2020). Given that all stop codons (TGA, TAA, and TAG) contain adenine nucleotides, ABE technology can be applied to correct nonsense mutations. We previously showed that this highly efficient gene editing tool converted A→G at the *CFTR* ^R553X^ locus when delivered as ribonucleoproteins (Krishnamurthy et al., 2021). In this study, we use AAV2.5T and AAV6.2 to deliver ABEs targeting the R553X locus of *CFTR* in primary human airway epithelial (HAE) cells. Since the total cargo size breaches the 4.7 kb packaging capacity of AAV, a split-intein system is employed to deliver the ABE. Successful editing was confirmed by high-throughput sequencing (HTS).

In this study, we generated and tested new AAV capsids designed by incorporating reciprocal point mutations to increase transduction efficiencies in airway epithelia. These capsids have widespread applications for lung delivery, including gene addition and gene editing approaches.

## 2 Materials and methods

### 2.1 AAV vector production

AAV capsids were designed *in silico* and synthesized by GenScript (Piscataway, NJ). AAV cassettes include CMV-eGFP, CMV-mCherry, or CBh driving expression of the N-terminal editing construct: adenine base editor and N-terminal half of Cas9^D10A^ ending with NpuN split-intein or C-terminal editing construct: C-terminal half of Cas9^D10A^ and the guide RNA targeting R553X. AAV vectors were produced and titrated as a fee for service at the University of Iowa Viral Vector Core (medicine.uiowa.edu/vectorcore/).

### 2.2 Human airway epithelial cells

The University of Iowa *In Vitro* Models and Cell Culture Core cultured and maintained HAE, as previously described (Karp et al., 2002). In brief, epithelial cells were dissociated from tracheal and bronchial tissues and seeded onto collagen-coated, semipermeable polycarbonate membranes with a pore size of 0.4 μm (surface area = 0.33 cm^2^; Corning Costar Transwells, Cambridge, MA). HAE remained submerged in Ultroser G (USG) medium for 24 h at 37°C and 5% CO_2_. After 24 h, the apical USG medium was removed, and the cells were incubated at the air–liquid interface. All cultures used for the experiments described were >3 weeks old and maintained a trans-epithelial electrical resistance of ≥500 Ω μm (Moss et al., 2007). Basal cells were cultured in Lifeline BronchiaLife media (Lifeline Cell Technology, Carlsbad, CA) for 3–5 days following lung harvest. When the cells reached 80% confluency, they were washed in phosphate-buffered saline (PBS), lifted in TrypLE (Gibco, Gaithersburg, MD), and seeded onto collagen-coated 24-well plates and transduced with each AAV capsid.

### 2.3 Transduction of HAE and basal cells

Airway epithelia were pretreated with the proteasome inhibitors doxorubicin (5 µM) and LLnL (40 µM) for 2 h (airway cultures) or 1 h (basal cells) prior to vector delivery. Apical transduction was accomplished by delivering a 50 μL mixture of serum-free medium and an MOI = 10,000 of each AAV capsid overnight to the apical surface of epithelial cells. For basolateral delivery, transwell cultures were inverted, and the basolateral surface was covered with 50 μL of each AAV capsid (MOI = 10,000) of serum-free medium and vector. Cells were incubated for 4 h at 37°C and 5% CO_2_ before the inoculum was removed, and the basolateral cultures were returned to their upright position. For the proteasome inhibitor experiment, airway cultures were treated overnight with 0, 0.1, 0.5, or 1 µM doxorubicin in the basolateral media, while AAV-mCherry vectors were delivered apically (MOI = 100,000) and replaced with fresh media the next day. Cells were imaged via fluorescence microscopy, and mCherry expression was quantified via flow cytometry.

### 2.4 Fluorescence microscopy and flow cytometry

GFP and mCherry images were acquired using a Keyence All-in-One Fluorescence Microscope BZ-X series (Osaka, Japan). Transwells were imaged at ×4 magnification. Three weeks following transduction of the cultures, GFP and mCherry levels were quantified via flow cytometry. In brief, cells were stained using a LIVE/DEAD Fixable Stain (Thermo Fisher Scientific, Waltham, MA), lifted in Accutase at 37°C for 30 min, and run through an Attune NxT Flow Cytometer (Thermo Fisher Scientific, Waltham, MA). The percentages of GFP^+^ cells were calculated. Cells were treated with Foxp3/Transcription Factor Staining Buffer Set (Thermo Fisher Scientific, Waltham, MA) according to the manufacturer’s recommendations and stained for 1 h at 4°C using the following antibodies : NGFR for basal cells (345110; 1:600, BioLegend, San Diego, CA), CD66c for secretory cells (12-0667-42, 1:1000, Invitrogen, Waltham, MA), and α-tubulin for ciliated cells (NB100-69AF405, 1:300, Novus, Centennial, CO). Cells were run on the Attune NxT Flow Cytometer, and expression was gated on live cells.

### 2.5 High-throughput sequencing

HTS was performed as previously described by Krishnamurthy et al. (2021). In brief, genomic DNA was isolated using the QuickExtract (#QE09050, Lucigen, Middleton, WI) according to the manufacturer’s protocol. The genomic sites of interest were PCR-amplified (KAPA DNA polymerase, Roche, Basel, Switzerland) with Illumina adapters flanked on the primers, followed by a secondary PCR. Purified PCR products were quantified and then sequenced using a single-end read of 200–250 bases on the Illumina MiSeq instrument using the manufacturer’s protocols. Base editing frequencies were further assessed using a previously described MATLAB script (Komor et al., 2016). For the results obtained for the human R553X mutation, the airway epithelia were compound heterozygous for this *CFTR* mutation. The allelic editing frequency was calculated using the following equation: (% editing − 50)/50. HTS data were deposited in the NIH Sequence Read Archive (SRA) database and are accessible under accession number PRJNA1027344.

### 2.6 Statistics

Student’s two-tailed *t*-test, one-way analysis of variance (ANOVA) with Tukey’s multiple comparison test, or two-way ANOVA with Dunnett’s multiple comparison test were used to analyze differences in mean values between groups. Results are expressed as mean ± SEM. *p-*values <0.05 were considered significant.

## 3 Results

### 3.1 Capsid modification design and rationale

AAV2.5T and AAV6.2 are two capsids with improved airway transduction properties compared to their respective parental serotypes. AAV2.5T is comprised of portions of the AAV2 and AAV5 capsids with a point mutation at position A581T (Table 1). AAV6.2 is identical to AAV6, except for an F for L substitution at position 129. We hypothesized that incorporating the A581T mutation into AAV6.2 might enhance its transduction properties in well-differentiated epithelial cells. Likewise, incorporating the F129L mutation into AAV2.5T might be similarly beneficial. Importantly, the A581 residue is conserved in AAV6.2, and the F129 residue is conserved in AAV2.5T. We generated capsids with reciprocal mutations (termed AAV2.5T^L^ and AAV6.2^T^) (Table 1).

**TABLE 1:** List of mutations, titers, and relevant citations for lung-tropic capsids. vg, vector genomes.

Capsid	Capsid modification	Titer (vg/ml)	Citation
AAV2		1.3 × 10^13^	Rabinowitz et al. (2002)
AAV5		1.1 × 10^13^	Chiorini et al. (1999)
AAV2.5T	A581**T**	2.1 × 10^13^	Excoffon et al. (2009)
AAV2.5T^L^	F129**L**/A581**T**	5.1 × 10^12^	
AAV2.5T^FF^	Y437**F**/A581**T**/Y720**F**	6.6 × 10^12^	
AAV2.5T^FF+L^	F129**L**/Y437**F**/A581**T**/Y720**F**	5.4 × 10^12^	
AAV6		2.8 × 10^12^	Halbert et al. (2001)
AAV6.2	F129**L**	1.0 × 10^12^	Limberis et al. (2009)
AAV6.2^T^	F129**L**/A581**T**	1.0 × 10^12^	
AAV6.2^FF^	F129**L**/Y445**F**/Y731**F**	4.3 × 10^12^	van Lieshout et al. (2018)
AAV6.2^FF+T^	F129**L**/Y445**F**/A481**T**/Y731**F**	7.6 × 10^11^	

**FIGURE 1:**
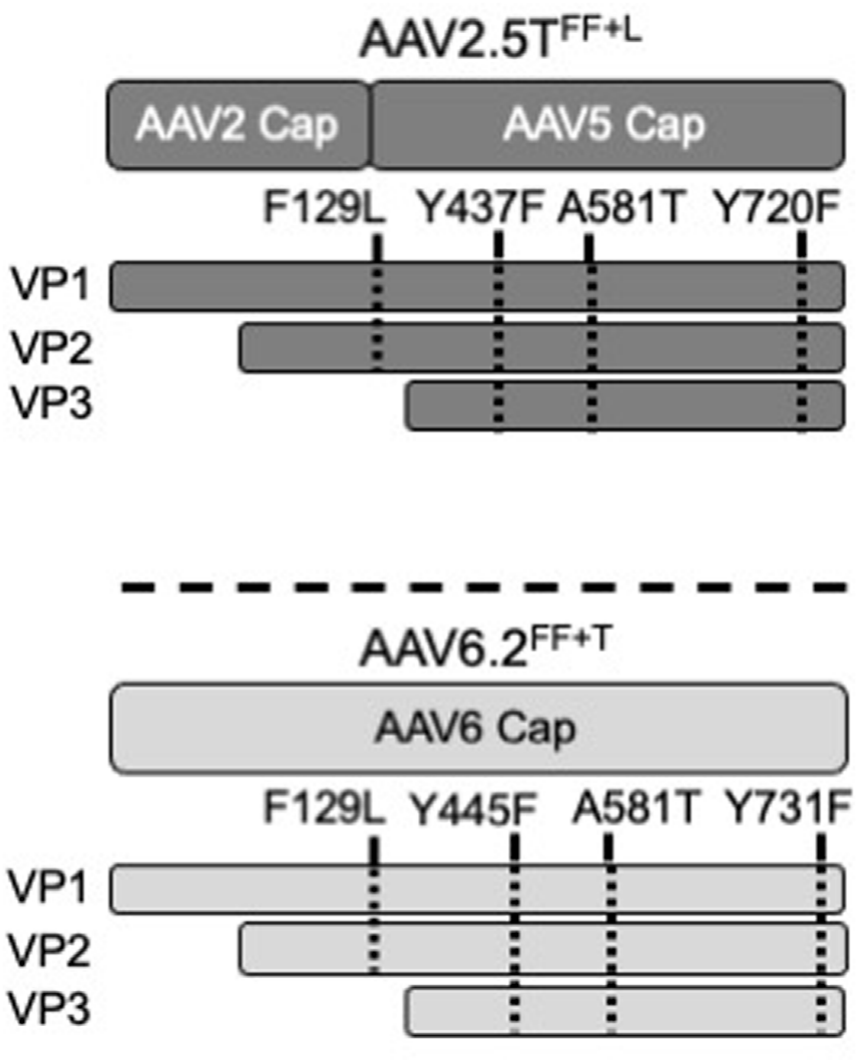
Schematic of AAV capsids. The locations of modified residues in VP1 or VP3 of AAV2.5T^FF+L^ and AAV6.2^FF+T^ capsids are shown. F129 and A581 are conserved between AAV2.5T and AAV6.2. Y437 and Y720 of AAV2.5T were chosen for modification because of their local similarity to Y445 and Y731 of AAV6.2.

Wootton and others reported that mutating two surface tyrosine residues of AAV6.2 to phenylalanine (Y445F and Y731F; termed AAV6.2FF) increased AAV internalization (van Lieshout et al., 2018). For consistency of nomenclature in these comparative studies, we designated this vector as AAV6.2^FF^. In addition, we generated analogous surface Y-to-F mutations (Y437F and Y720F) in AAV2.5T (termed AAV2.5T^FF^) to determine if they similarly enhanced airway tropism in a different capsid (Table 1). Lastly, we generated the combinatorial mutations AAV2.5T F129L, Y437F, and Y720F (termed AAV2.5T^FF+L^) and AAV6.2L A581T, Y445F, and Y731F (termed AAV6.2^FF+T^) (shown schematically, Figure 1).

### 3.2 Transduction properties of capsid modifications on well-differentiated human airway epithelia

**FIGURE 2:**
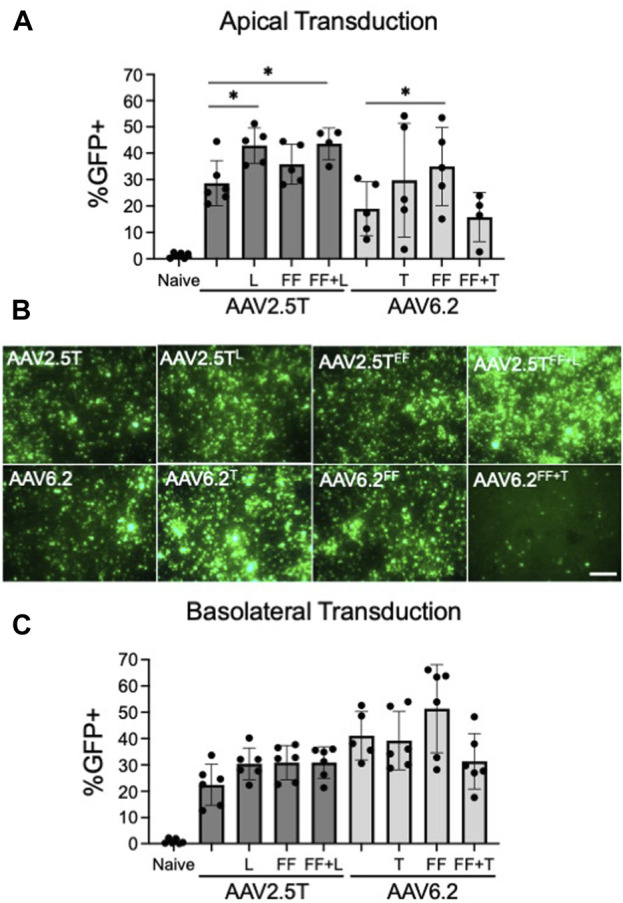
Transduction efficiencies of AAV2.5T and AAV6.2 capsid variants on primary human airway epithelia. **(A)** Cells were transduced apically overnight with the indicated AAV capsid delivering CMV-eGFP (MOI = 10,000). Two weeks post-transduction, GFP expression in airway cultures was quantified via flow cytometry. Naïve cells were untransduced. **(B)** Representative low-power fluorescent images from cultures in **(A)**; scale bar = 1 mm. **(C)** Airway cultures were inverted and transduced with the indicated capsid delivering CMV-eGFP (MOI = 10,000) on the basolateral surface for 4 h. Following the vector application, the transwells were returned to their upright position. GFP expression was quantified after 2 weeks via flow cytometry. *n* = 6 human donors; *p < 0.05.

To test the ability of each capsid variant to transduce airway epithelia, we assessed gene transfer from each capsid on well-differentiated human airway epithelial cells. We pre-treated HAE with proteasome modulators doxorubicin (5 µM) and LLnL (40 µM) for 2 h, followed by an overnight transduction (MOI = 10,000). We applied vector encoding a CMV-GFP expression cassette to the apical surface of well-differentiated primary cultures grown at an air–liquid interface on transwell inserts. The percentage of GFP^+^ cells was quantified via flow cytometry 2 weeks post-transduction (Figure 2A). AAV2.5T^L^ and AAV2.5T^FF+L^ conferred increased %GFP expression compared to the parental AAV2.5T. AAV2.5T^FF^ trended toward increased expression but did not reach statistical significance. Of the AAV6.2 variants, only AAV6.2^FF^ conferred a statistically significant increase in % GFP expression over the parental capsid (Figure 2A). Of note, AAV6.2^FF+T^ had reduced vector titers (Table 1), suggesting that these combinatorial mutations may be detrimental to vector production. Representative fluorescence images of transduced HAE using each of the capsid variants are shown (Figure 2B).

We next asked if vector delivery to the basolateral surface of HAEs would increase transduction. For basolateral delivery, HAE cultures were inverted, and vectors were applied to the basolateral surface for 4 h. The transwells were then returned to their upright position. The percentage of GFP^+^ cells was quantified via flow cytometry 2 weeks post-transduction (Figure 2C). For the three AAV2.5T capsid variants, there was no statistically significant increase in the % GFP^+^ cells over the parental capsid. Of the AAV6.2 capsid variants, only AAV6.2^FF^ showed a trend for a greater percentage of GFP^+^ cells than its parental capsid but did not reach statistical significance (Figure 2C). We conclude that the tested capsid modifications do not enhance basolateral transduction.

### 3.3 Cell types transduced following apical or basolateral vector delivery

**FIGURE 3:**
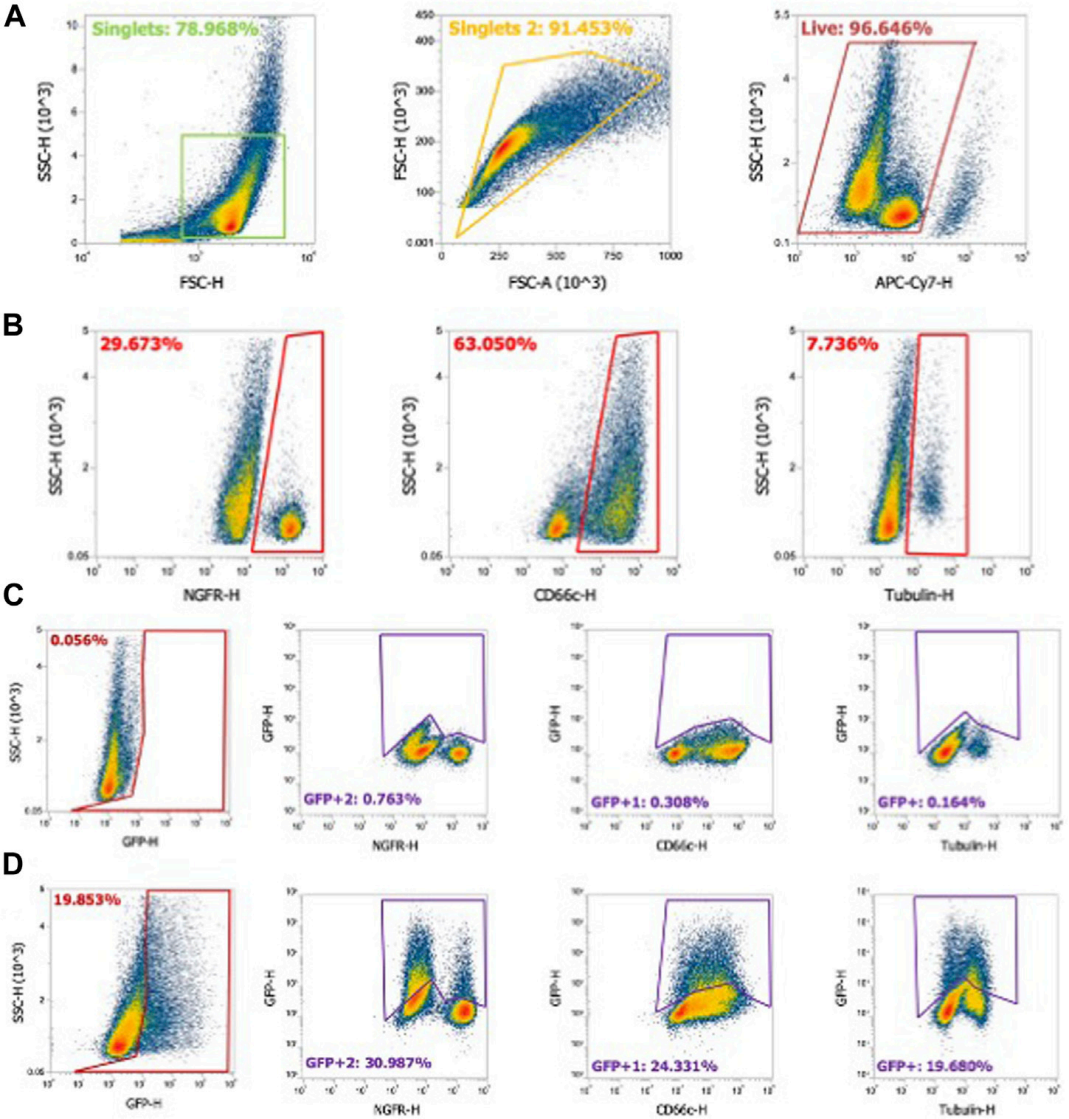
Flow cytometry gating strategy for airway epithelial cell types. Human airway epithelial cultures were stained for cell types as described in Materials and methods. **(A)** Using flow cytometry, cells gated on singlets were first gated as forward scatter height (FSC-H) by side scatter height (SSC-H) and further gated by forward scatter area (FSC-A) by FSC-H. Live cells stained with fixable LIVE/DEAD near-IR stain were gated as shown. **(B)** NGFR (basal cells), CD66c (secretory cells), and α-tubulin (ciliated cells) were gated from the cell population. **(C)** Gating strategy for naïve airway epithelia for GFP-negative cells. Panel 1: GFP gating on the whole cell population. Panel 2–4: GFP gating strategy for NFGR (basal cells), CD66c (secretory cells), and α-tubulin (ciliated cells). **(D)** Example of gating in GFP^+^ airway epithelia and GFP^+^ cell types.

Generating single-cell suspensions and performing flow cytometry with differentiated airway–liquid interface primary airway epithelial cultures presents greater challenges compared to traditional cell culture models. First, single cells are defined using two approaches: forward scatter height (FSC-H) by side scatter height (SSC-H) and further gated as forward scatter area (FSC-A) by FSC-H (Figure 3A). Live cells expressing the fixable LIVE/DEAD stain are defined. Basal cells, secretory cells, and ciliated cells comprise the majority of cell types in HAEs. A flow cytometry strategy using αNGFR (basal cells), αCD66c (secretory cells), and α-acetylated alpha-tubulin (ciliated cells) provides an estimate of the cell-type distribution ratios (Figure 3B). To quantify the transduced GFP^+^ cell types, gates are first defined in naïve HAEs (Figure 3C), subsequently quantifying the shift in GFP^+^ cells following transduction (Figure 3D). Representative gates of the shift of GFP expression in the entire population (first panel) and individual cell types (second–fourth panels) are shown.

**FIGURE 4:**
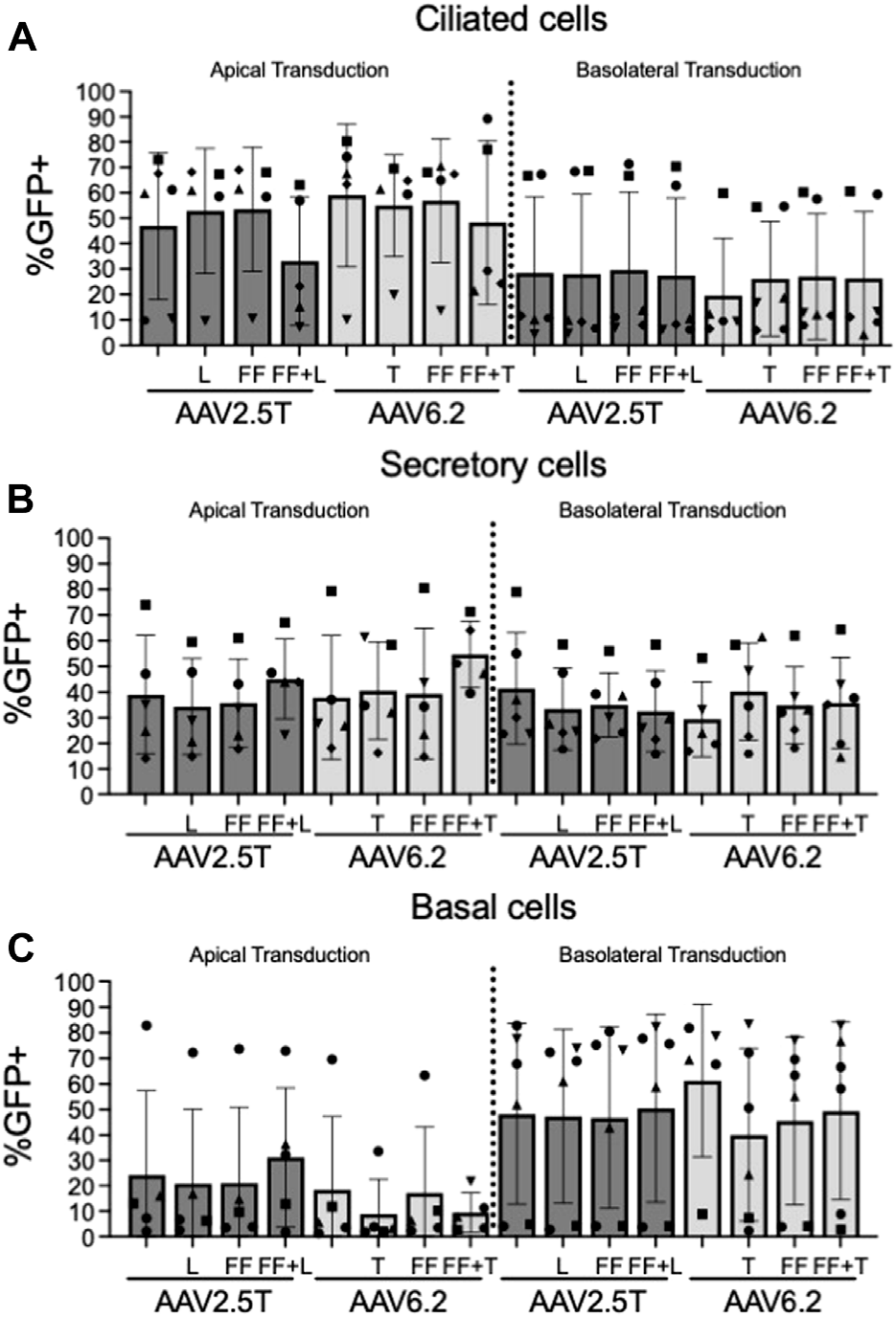
Cell types transduced by each AAV capsid. Airway epithelia were transduced from the apical or basolateral surface with each AAV capsid, as described in Figure 2. Airway cultures were lifted in Accutase and labeled for **(A)** ciliated cells (α-tubulin), **(B)** secretory cells (CD66c), and **(C)** basal cells (NGFR) cellular markers, as described in Figure 3. Cell types were gated from the GFP^+^ population. *n* = 6 human donors.

We next quantified the cell types transduced for each of the AAV capsid variants following apical or basolateral application of the AAV vector expressing eGFP. As described in Figure 2, AAV was delivered to either the apical side overnight or the basolateral surface for 4 h. Two weeks post-transduction, the cell types transduced were quantified via flow cytometry (Figures 4A–C). The flow cytometry gates were set to evaluate the cell types transduced in the GFP^+^ population. Thus, although the levels of transduction vary between capsids, as shown in Figure 2, the overall ratios of cell types transduced do not vary, suggesting that altering the capsids does not change the transduction preference for each cell type. Additionally, we conclude that the capsid modifications do not alter cell type tropism as compared to its parental capsid. The ratio of cell types varies from donor to donor, which contributes to an increased experimental error when quantifying vector-conferred cell tropism. Ratios of cell types transduced were calculated by normalizing to parental capsid distribution (Table 2). The percentage of cell types transduced was largely consistent between the AAV2.5T- and AAV6.2-based vectors. As anticipated, basolateral application of the vector skewed the ratios toward basal cells. Although not shown, the mean fluorescent intensity of reporter gene expression was similar for all capsid variants.

**TABLE 2:** Summary of cell types transduced by all parental and variant capsids. Average % GFP^+^ cells ± standard error of all AAV2.5T and AAV6.2 variants combined from apical and basolateral applications. Each cell type represents the average of all data points in Figure 4.

	Capsid combination	Ciliated	Secretory	Basal
Apical delivery	AAV2.5T (including L, FF, and FF + L)	53% ± 5.8%	38% ± 4.5%	22% ± 6.6%
	AAV6.2 (including T, FF, and FF + T)	50% ± 6.7%	42% ± 4.9%	24% ± 7.1%
Basolateral Delivery	AAV2.5T (including L, FF, and FF + L)	26% ± 5.7%	33% ± 2.9%	51% ± 6.8%
	AAV6.2 (including T, FF, and FF + T)	26% ± 5.4%	37% ± 3.9%	45% ± 7.6%

### 3.4 Transduction properties of capsid mutations in primary human airway basal cells

**FIGURE 5:**
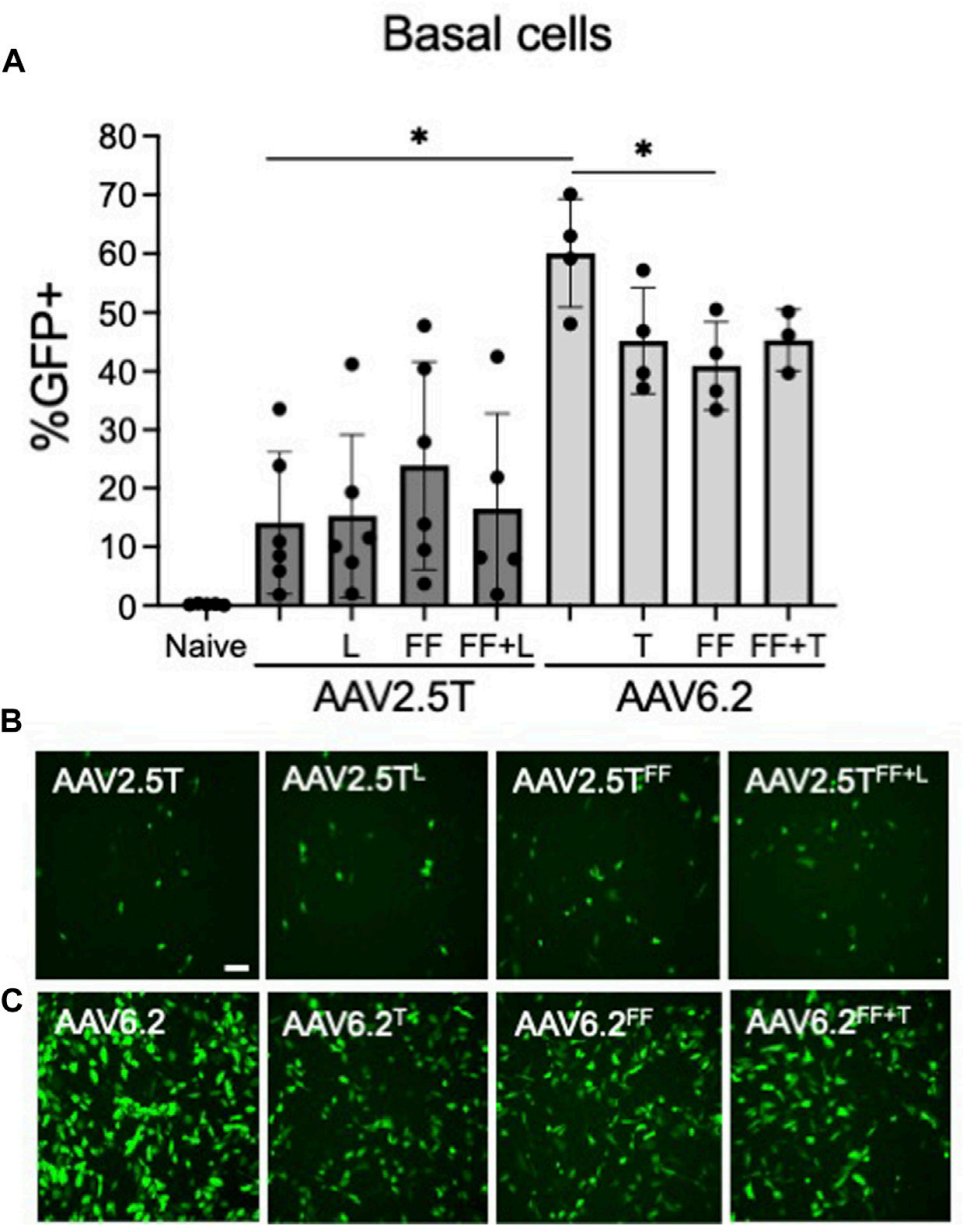
Primary airway basal cell transduction profile by AAV capsids. Primary basal cells were seeded on collagen-coated plates. One day post-seeding, the indicated AAV capsid variants delivering CMV-eGFP (MOI = 10,000) were applied for 4 h **(A)** GFP expression was quantified 2 days post-transduction via flow cytometry. Representative images of **(B)** AAV2.5T-based capsid variants and **(C)** AAV6.2-based capsid variants are shown. Scale bar = 2 mm. *n* = 6 human donors; *p < 0.05.

Basal cells lie on the basement membrane of surface airway epithelia and are progenitor cells of the conducting airways, which replenish terminally differentiated cells following cell turnover. Transducing basal cells with an AAV gene therapy reagent will prolong therapeutic transgene expression. Primary basal cells are dissociated directly from human lung donor tissue and seeded onto tissue culture plates. The cells are then immediately transduced before they differentiate. We previously reported that at the time of seeding, 95% of primary basal cells were positive for basal cell markers (Li et al., 2019). We asked if AAV2.5T or AAV6.2 capsid variants had improved basal cell tropism compared to parental capsids. AAV2.5T^FF^ was marginally improved over AAV2.5T but did not reach statistical significance (Figure 5). Overall, AAV6.2 showed the greatest basal cell transduction efficiency. Unexpectedly, additional capsid modifications of AAV6.2 resulted in decreased primary basal cell transduction.

### 3.5 Proteasome modulation with “FF” capsid mutants

**FIGURE 6:**
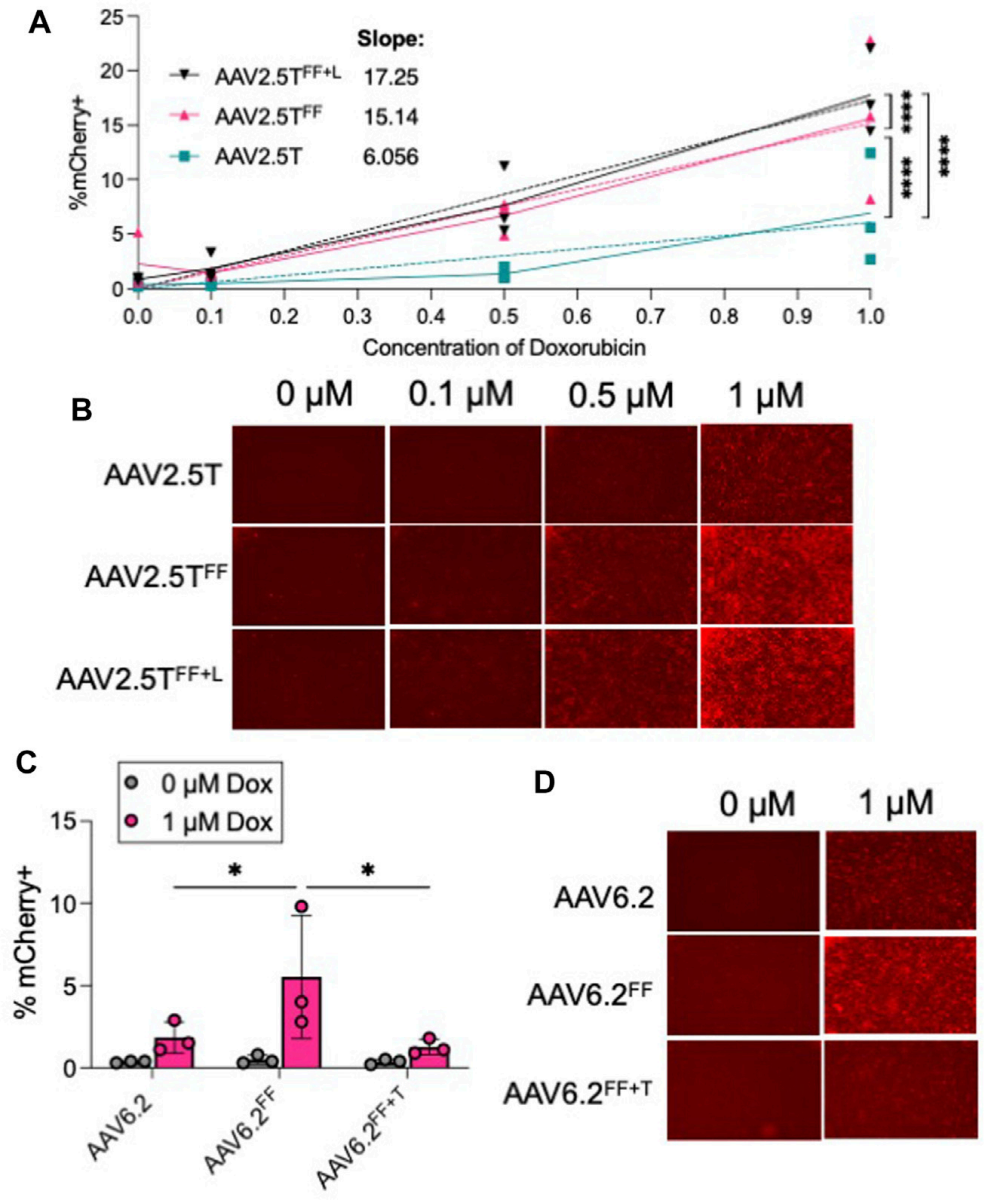
Proteasome inhibitor dose response to “FF” containing AAV capsids. **(A)** Well-differentiated airway epithelia were treated with doxorubicin (Dox) in the basolateral media (0, 0.1, 0.5, and 1 µM), and AAV2.5T, AAV2.5T^FF^, or AAV2.5T^FF+L^ expressing mCherry (MOI = 100,000) was applied to the apical surface overnight. Two weeks later, mCherry expression was quantified via flow cytometry. **(B)** Representative images from each condition. **(C)** Airway cultures were treated with 0 or 1 µM Dox in the basolateral media along with AAV6.2, AAV6.2^FF^, or AAV6.2^FF+T^ (MOI = 100,000) on the apical surface overnight. Two weeks later, mCherry expression was quantified via flow cytometry. **(D)** Representative images for each condition. *n* = 3 human donors; *p < 0.05 and ****p < 0.00005.

The rationale for incorporating “FF” residues into AAV capsids was to increase transduction by reducing ubiquitinated tyrosine residues that are typically shuttled to the proteasome for degradation (Zhang et al., 2004). We hypothesized that incorporating the Y to F residue change would reduce the need for a proteasome inhibitor. We compared the transduction efficiencies of AAV2.5T, AAV2.5T^FF^, and AAV2.5T^FF+L^ (MOI = 100,000) in the presence of 0, 0.1, 0.5, and 1 µM Dox (Figures 6A, B). For each AAV2.5T variant tested, we observed that increasing Dox concentrations improved transduction, but the “FF” capsids still required Dox to transduce airway epithelia. However, the steeper dose–response slope of the AAV2.5T^FF^ and AAV2.5T^FF+L^ variants suggests that lower concentrations of Dox have a more potent impact on gene expression compared to the parental capsid. For AAV6.2, AAV6.2^FF^, and AAV6.2^FF+T^, we compared the presence (1 µM) or absence (0 µM) of Dox (Figures 6C, D). We observed no increase in transduction of the “FF” in the absence of Dox, suggesting that the increase in transduction observed with AAV6.2^FF^ is due to a mechanism independent of proteasome modulation by Dox. Overall, incorporating the “FF” residues in the AAV2.5T capsid improved transgene expression compared to the parental capsid at each Dox concentration tested.

### 3.6 Genomic editing following AAV-mediated ABE delivery

**FIGURE 7:**
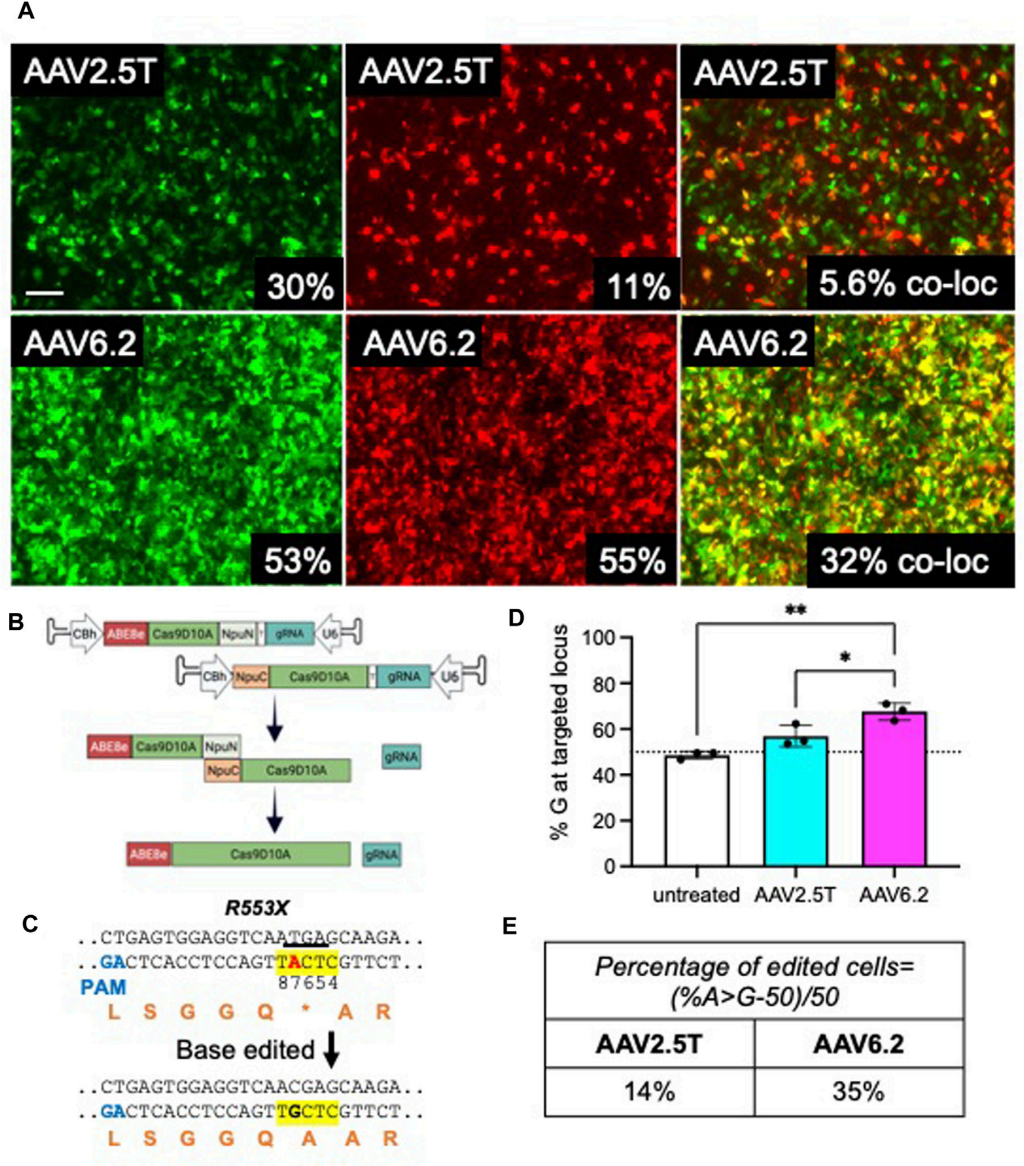
Adenine base editors repair the premature stop codon in R553X CF HAEs. Well-differentiated CFTR^R553X/L671X^ airway cultures were lifted and reseeded to enhance basal cell transduction. A day after lifting, cells were co-transduced with AAV2.5T-CMV-eGFP + AAV2.5T-CMV-mCherry or AAV6.2-CMVe-GFP + AAV6.2-CMV-mCherry (total MOI = 10,000) overnight. **(A)** Two weeks post-transduction, airway cultures were imaged and lifted, and GFP^+^, mCherry^+^, and dual-positive cells were quantified via flow cytometry. Scale bar = 1 mm. Flow cytometry quantification is shown in the bottom right of each image. **(B)** Parallel cultures received AAV2.5T or AAV6.2 N-terminal and C-terminal ABE/Cas9^D10A^ with a gRNA targeting the R553X locus of *CFTR* (total MOI = 10,000) as shown schematically. **(C)** The sequence of the R553X locus of *CFTR* is shown. The yellow highlight indicates the ABE editing window. Red “A” indicates the ABE target. The underline indicates the “TGA” stop codon of R553X. Orange letters are amino acid residues. PAM, protospacer adjacent motif. **(D)** High-throughput sequencing data quantifying percent adenine-based editor-mediated conversion of A→G. **(E)** Percentage of edited cells formula and percentages reported. The cells were compound heterozygous for the *CFTR* ^R553X^ mutation. *n* = 3 technical replicates from one CF lung donor; *p < 0.05 and **p < 0.005.

Because the size of ABE using *Streptococcus pyogenes* Cas9 exceeds the packaging capacity of AAV vectors, typical AAV-CRISPR-based ABE gene editing strategies require co-expression from two AAVs in the same cell (Levy et al., 2020). In this study, we determine the co-transduction efficiency of two AAVs in HAEs using reporter genes. In efforts to transduce progenitor cells, cells from a transwell filter were lifted in TrypLE for 30 min and reseeded. Twenty-four hours post-seeding, CF cells were pre-treated with doxorubicin (5 µM) and LLnL (40 µM) for 1 h prior to transduction. AAV2.5T CMV-GFP + AAV2.5T CMV-mCherry or AAV6.2 CMV-GFP + AAV6.2 CMV-mCherry were co-transduced at a total MOI = 10,000 overnight. Cells were imaged via fluorescence microscopy, and GFP and mCherry expression was quantified via flow cytometry 2 weeks later (Figure 7A). Using this delivery protocol, AAV6.2 achieved more co-transduction (32%) than AAV2.5T (5.6%).

We previously reported efficient editing of the *CFTR* ^R553X^ locus in primary cells by electroporating ABE8e ribonucleoproteins (RNPs) (Krishnamurthy et al., 2021). In this study, we used AAV2.5T and AAV6.2 to deliver ABE8e using a previously described split-intein approach (Levy et al., 2020). As shown, the ABE was divided between two AAV vectors (Figure 7B). The full-length ABE is stitched by an NpuN and NpuC split-intein in the target cells. We tested this delivery system in CF primary human airway epithelia carrying CFTR^R553X^ and CFTR^L671X^ alleles. Using ABEs targeted to correct the R553X variant of *CFTR* (Figure 7C), we co-delivered the AAV-ABE N-term and AAV-ABE C-term vectors. Using the same delivery protocol described in Figure 7A, a vector was delivered to HAEs derived from a human lung donor carrying the *CFTR* ^R553X/L671X^ genotype. Two weeks post-transduction, we quantified the editing efficiency using high-throughput sequencing and measured the percentage of A→G conversion at the R553X locus and the percentage of edited cells (Figures 7D, E). Consistent with the observed levels of reporter gene co-transduction, we observed an allelic editing efficiency of ∼14% for AAV2.5T-ABE vectors and ∼35% for the AAV6.2-ABE vector. In conclusion, AAV is an efficient tool for ABE delivery to airway cells, and capsid choice may play an important role in delivery efficacy.

## 4 Discussion

The AAV capsids generated in this study were designed by rational incorporation of residues hypothesized to improve the transduction efficiency of airway epithelial cells relative to the parental airway tropic AAV2.5T and AAV6.2 capsids. In this study, we observed that AAV2.5T better transduces well-differentiated HAEs compared to AAV6.2; however, AAV6.2 demonstrated greater transduction of airway progenitor cells. This is consistent with our previous screening of 10 AAV candidates expressing GFP on primary basal cells. In that study, AAV1, AAV2, and AAV6 all transduced primary human basal cells, but AAV6.2 resulted in the highest percentage of GFP^+^ cells (Brommel et al., 2020). We anticipated that combinatorial mutations would enhance transduction properties for airway surface epithelial cells and/or basal cells. Our results suggest increased transduction efficacy using the AAV6.2^T^ and AAV6.2^FF^ variants over the parental AAV6.2. In contrast, we observed that AAV6.2^FF+T^ had the lowest vector titer, suggesting that a threshold in AAV6 capsid modification may have been reached. In addition, we observed that the transduction efficiency of AAV6.2^FF^ matched AAV2.5T following apical transduction. The capsid with the highest transduction efficiency from the apical surface and the highest titer was AAV2.5T^FF+L^.

Possible immune responses to AAV include 1) neutralization by pre-existing anti-capsid antibodies, 2) TLR2 recognition at the cell surface, 3) TLR9 recognition of single-stranded DNA, 4) proteasomal degradation of capsid proteins, and 5) humoral and cytotoxic T-cell-mediated clearance (reviewed in Ronzitti et al. (2020)). Modifying AAV capsids is a strategy to reduce toxicity. We and others reproducibly observe that proteasome inhibitors can greatly increase transgene expression from AAV vectors (Ding et al., 2003; Mitchell and Samulski, 2013). After cell entry, surface tyrosine residues in the AAV capsid can be ubiquitinated, causing the vector to be shuttled to the proteasome for degradation (Zhong et al., 2008a). Thus, the inclusion of specific Y-to-F capsid mutations is postulated to further improve airway transduction by de-targeting the vector from proteasomal degradation and improving access to the nucleus (Kang et al., 2020). This strategy has been investigated in AAV serotypes 2, 6, 6.2, 8, and 9, each leading to increased transduction in various tissues (Zhong et al., 2008b; Markusic et al., 2010; van Lieshout et al., 2018). The AAV6.2 variant used in this study (AAV6.2^FF^) with Y-to-F mutations at positions Y445 and Y731 was previously reported by Wootton and others (van Lieshout et al., 2018). Here, we show that the “FF” variants generated for this study still require Dox for transgene expression. However, incorporating “FF” mutations into AAV2.5T increased transduction efficiencies relative to AAV2.5T at each Dox concentration tested. AAV6.2 “FF” variants did not enhance transduction in the absence of proteasome modulation, suggesting a proteasome-independent mechanism for increasing transduction.

Gene therapy using gene addition or genome editing approaches is advancing at a rapid pace. Still, an effective airway delivery approach remains a bottleneck. ABEs are an innovative technology to introduce A→G edits without the need for double-stranded breaks or cell division. AAV is a promising delivery vehicle to the lung but is limited by its packaging capacity. Employing a split-intein system in which two AAVs can deliver N- and C-terminal cargo fused by the inteins is a strategy to overcome the size limit. In this study, we compared AAV2.5T and AAV6.2 to deliver ABEs using split inteins to CF HAEs and observed 14% and 35% allelic editing efficiency, respectively. Given the rare opportunity to test a CF cell donor with this mutation, only AAV2.5T and AAV6.2 were tested since the other capsids carrying the split intein ABEs had not been produced yet when this tissue was available. AAV-transduced epithelia did not form an electrically tight layer suitable for measurements of bioelectric properties, and therefore, anion channel activity could not be quantified using the Ussing chamber. Future studies will include delivering the ABEs using a single AAV, aiming to enhance editing efficiencies compared to dual AAV deliveries.

A barrier to advancing either gene addition or gene editing strategies for CF is achieving efficacy from an aerosolized administration in the lung. Finding a capsid that would increase airway transduction and decrease the need for proteasome inhibitors is an important stepping stone to advancing a gene therapy treatment for CF. While AAV2.5T, AAV6.2, and AAV6.2^FF^ have improved transduction, incorporating residues from AAV6.2 and AAV6.2^FF^, the AAV2.5T^FF+L^ may improve the therapeutic index, increase transduction efficiencies, and reduce the need for proteasome inhibitors.

## Data Availability

The datasets presented in this study can be found in online repositories. The names of the repository/repositories and accession number(s) can be found at: https://www.ncbi.nlm.nih.gov/; PRJNA1027344.
